# The Role of 3D Exoscope Systems in Neurosurgery: An Optical Innovation

**DOI:** 10.7759/cureus.15878

**Published:** 2021-06-23

**Authors:** Brian Fiani, Ryan Jarrah, Daniel W Griepp, Jessica Adukuzhiyil

**Affiliations:** 1 Neurosurgery, Desert Regional Medical Center, Palm Springs, USA; 2 Neurosurgery, College of Arts and Sciences, University of Michigan - Flint, Flint, USA; 3 Neurosurgery, College of Osteopathic Medicine, New York Institute of Technology, Old Westbury, USA; 4 Medicine, College of Osteopathic Medicine, New York Institute of Technology, Old Westbury, USA

**Keywords:** exoscope, microscope, illumination, visualization, optics, neurosurgical technology, operative field, 3-dimensional

## Abstract

The development of the three-dimensional (3D) exoscope is a marvel of technological innovation in modern surgical practice. While its predecessor, the operating microscope (OM), has long been the gold-standard surgical visualization modality, its particular limitations in terms of accessibility and ergonomic demand have led to the development of a more sophisticated, 3D model. Specifically, the 3D exoscope allows for an enhanced image quality of the surgical field, while also being more ergonomically favorable. Moreover, this device’s ability to handle delicate microsensitve procedures, along with its alleviation of surgeon fatigue, indicates great potential for neurosurgical application. For this narrative review, the authors queried PubMed database using the keyword “exoscope” to identify relevant studies involving the specialty of neurosurgery that were published in English language full text. The search yielded full-text English language-related articles regarding neurosurgical exoscope, its applications and limitations. The 3D exoscope uniquely allows for enhanced surgeon comfort and superior imaging of the patient’s real-time anatomy. However, the OM was described to having a slight image favorability with fusion and decompression surgery. Cost analysis is highlighted for its potential disparity. 3D exoscopes will potentially be incorporated with intelligent carriers and robotic surgical systems. Ultimately, with further studies highlighting its use, the 3D exoscope is expected to continue to imprint its status as one of the most efficient technological visualization tools in the future of neurosurgical practice.

## Introduction and background

The incorporation of visual enhancement technology has transformed the field of neurosurgery. First introduced in the late 1950s, the operating microscope (OM) became the gold standard in neurosurgery for intraoperative visualization due to its ability to illuminate and magnify the surgical anatomy [1,2]. Despite these advances, the OM was found to have limitations in operative mobility, accessibility and expense. Additionally, the operational view of the OM is solely limited to the operating and assisting surgeons, while also forcing the surgeons to maintain an uncomfortable posture and thus potentially promoting intraoperative fatigue [2]. To ameliorate these shortcomings, while preserving the advantages of the OM, the extracorporeal telescope (exoscope) was recently created [3]. The exoscope system is composed of a scope positioned outside of the body cavity such that a high-definition (HD) view of the surgical field is projected onto a two-dimensional (2D) or 3D high-resolution monitor. Recent efforts to preserve stereopsis as well led to the development of the 3D exoscope system [4]. The gradual application of the 3D exoscope system in a multitude of neurosurgical procedures such as cranial and spinal surgeries has suggested that it is a safe and effective alternative to the OM [3,5,6]. A few clinical studies have demonstrated that the 3D exoscope preserves illumination and magnification, while also being ergonomically more favorable [3,6,7]. Furthermore, the 3D exoscope has demonstrated high-quality visualization for all individuals present in the operating room, suggesting its potential to be an important tool in the education of those who are training [1,2,6,8,9]. The purpose of this study is to review the existing 3D exoscope systems and to characterize the clinical, functional and financial outcomes of the 3D exoscope, an uncommon, yet credible and versatile neurosurgical visualization tool.

## Review

Novel nuances

Multiple exoscope systems are currently available for neurosurgical use, including the VITOM® 3D (Karl Storz SE & Co. KG, Tuttlingen, Germany), KINEVO® (Carl Zeiss Meditec AG, Oberkochen, Germany), Modus V™ (Synaptive Medical, Toronto, Canada), and ORBEYE™ (Olympus, Tokyo, Japan) [2]. These next-generation exoscopes contain 4K-3D displays, light filters for 5-aminolevulinic acid and indocyanine video-angiography, pneumatic arms, adjustable operative settings, multiscreen output, longer focus distance and a greater magnification power [10]. Multiple clinical studies have recently used ORBEYE and VITOM 3D and thus these models’ device specifications will be reviewed in detail.

The ORBEYE 3D exoscope system was the first to have 4K HD clarity concurrently with 3D visualization [6]. It is composed of two metal-oxide semiconductor cameras, each having a resolution of 3840 x 2160 pixels. Each of the cameras has an individual counterbalance arm operated by a traditional dead man switch. The camera body itself contains the dead man switch and the controls for zoom and focus. The exoscope and the counterbalance arms are mounted on a console. The operating system for the exoscope is Unix-like and it is controlled by a graphical user interface established within the console itself. Illumination of the field is carried out by a fiber-optic LED source [9,11]. The system creates its image via a 3D line-by-line (LBL) mode that can be displayed in compatible 3D monitors that use the same resolution as the capturing cameras. The image created is a full-resolution circularly polarized passive 3D image that requires the observer to wear circularly polarized 3D glasses to properly view it [9,11]. Optical and digital zooming can be performed by either a hand or foot switch located on the exoscope. The magnification can range from 1.1 to 25.8 times, while using a zooming ratio of 1:12 times (six times via optical zooming and two times via digital zooming). This exoscope has a focal length of 220-550 mm and focus can be adjusted via the exoscope headpiece or by using a foot pedal. The field of view for the exoscope can range from 7.5 to 171 mm [9,11].

The Video Telescope Operating Microscope (VITOM) 3D includes 4K sensor system that is able to create an image with a resolution of 3840 x 2160 pixels, displayed onto two 4K 3D monitors. This system has a focal length of 200-500 mm and an adjustable zoom range that is able to magnify a field from as little as 8 times to 30 times. The IMAGE1 PILOT is a control unit mounted on a rotation socket and articulation stand that can operate the 3D exoscope such that adjustments can be made to redirect view to a region of interest, or simply to adjust zoom and focus [2,7]. Of note, other available models Modus V and KINEVO are alternative exoscopes for use and have similar parameters as the exoscope systems described above.

Neurosurgical application

Spinal Meningioma Surgery

Spinal meningiomas are common extramedullary spinal tumors that arise from the overexpression of meningothelial arachnoid cells within the thin membrane of the spinal membrane known as the dura [12,13]. They represent about two-thirds of all spinal neoplasm cases; however, they are less prevalent than their cranial analogs [14,15]. With the vast majority of spinal meningiomas being benign, this tumor’s symptomatic effects occur secondary to compression and mass effect on the spinal cord or nerve roots [13]. Common symptoms include difficulty in walking, pain at the tumor site, loss of urinary control, loss of sensations, extremity weakness, or neurological deficits [13]. Radiographic imaging shows the pathology is highly localized in the thoracic region, while surgery is noted to be the main line of treatment [13,16]. Surgery has been associated with favorable clinical outcomes, largely due to new methods of neuromonitoring [17].

Spinal meningiomas are an example of a pathology that can benefit from the 3D exoscope technology. With spinal meningiomas being mostly within the cervical or thoracic regions, a large surgical view is required because these regions present deeper operative fields [16,18]. The 3D exoscope ideally presents the operative environment illustrated broadly in a 3D landscape [18,19]. Meanwhile, the tumor itself is slow-growing and is quite small, presenting the need for optimal magnification and illumination of the epidural spinal canal, surrounding vessels, and tumor-dural attachment points [18]. As meningiomas do, they could spread across three-laminae layers, warranting for dynamic imaging adjustments. Moreover, spinal surgery often requires fluoroscopy, meaning the OM has to accommodate for the multiple surgical transitions [18]. The 3D exoscope easily accommodates for flexible image adjustments, while also not interfering with fluoroscopic placement, allowing for a better tumor dissection and an efficient surgical experience [18,19]. In addition, the large working distance, the camera holding arm being on the opposite side of the surgeon, the exoscope being at the center of the surgical field, with the monitor provided in front of the surgeon while using the 3D exoscope will eliminate conflict between surgical instruments, making the surgical setting more ergonomically favorable. Nevertheless, the greatest indications for the use of the 3D exoscope depend on the meningioma’s size, proximity to surrounding neurovascular components, and surgeon experience. Therefore, before conducting tumor removal, it is advised to consider an OM as an alternative [18,19].

Despite the clear benefit of using the 3D exoscope in spinal meningioma surgery, studies describing its usage are sparse [18]. A case report by de Divitiis et al. described that the 3D exoscope provided excellent image quality of the extradural and intradural space in spinal meningioma resection (Table 1) [18]. Moreover, the positioning of the camera never had to be adjusted, with the ability to zoom and focus being conducted mainly during the tumor dissection [18]. In another study by de Divitiis et al., two dorsal meningioma surgeries were conducted with the results again showing excellent resolution, with the need for readjustment only being noted when transitioning from epidural to intradural [19].

**Table 1:** Summary of studies using the 3D exoscope for neurosurgical application 3D: three-dimensional; ACDF: anterior cervical discectomy and fusion; LPD: lumbar posterior decompression; OM: operating microscope; LOS: length of stay

Study	Surgery	Major Finding
de Divitiis et al. [18]	Spinal meningioma surgery	The 3D exoscope provided excellent image quality of the extradural and intradural space; no need for readjustment
Vetrano et al. [11]	Peripheral nerve sheath tumor surgery	Enhanced surgeon comfortability interface between the pathological and surrounding tissue delineated and noted for image quality; no need for OM usage
Barbagallo & Certo [20]	ACDF	Allowed for easy maneuvering and surgical field adjustment; assisted with lateral and V-shape drilling; videos generated with surgeon operative view; no described complications
Siller et al. [2]	ACDF and LPD	No difference between the 3D exoscope and OM in terms of blood loss, operative time, LOS; 3D exoscope has significantly better surgeon comfort; image quality noted as slightly superior for ACDF surgery with an OM

Peripheral Nerve Sheath Tumor Surgery

Peripheral nerve sheath tumors (PNSTs) are also potential pathologies that can benefit from the 3D exoscope. PNSTs are rare, biologically aggressive type of tumors that occur along the lining, or sheath, of peripheral nerve cells [21-23]. They grow in the soft tissues of the body, such as muscle, fat, lymph, and blood vessels, and can quickly metastasize in the body [23,24]. Their pathogenesis typically expands from the spinal cord into the extremities of the body, and causes symptoms such as pain and weakness along the affected area [21]. However, while the pain-like symptoms are often predicted, the clinical course of PNSTs is variable based on the nerves involved along the affected injury. The pain-like symptoms are due to the invasion of the cancer into the nerves and surrounding tissue that causes the lesion to range in pain severity [25]. In addition, the tumor itself can occur sporadically or as a result of genetic anomalies such as neurofibromatosis types 1 and 2 or schwannomas [26,27]. The majority of PNSTs are benign, with surgery being the most common treatment modality [26].

Recently, advanced endoscopic techniques, such as the use of a 3D exoscope, have been used for PNST surgery. This surgery is meant to preserve functional cellular structures to provide a total resection of the tumor in a safe and effective way [11]. With the 3D exoscope’s capability of increasing the visualization of anatomical details, the layers of the tumor-nerve interface become more accessible for surgical removal. The traditional use of the OM has limitations for PNST surgery, as the physical structure of the microscope can be ergonomically unfavorable for the surgical experience, while the vision provided does not offer the quality that is needed for deep tissue precision. The 3D endoscope system, such as ORBEYE, provides 4K, multi-dimensional, and high-resolution imaging of the surgical field allowing for increased accuracy in tumor removal [11]. Meanwhile, procedures can be performed with neurophysiological monitoring to identify the functionality of nerves in the surrounding tissue [11]. The exoscope also comes with a supporting arm that helps allow better visualization of hidden angles associated with PNST surgery [11]. In addition, these exoscopes are supplied with specific light filters for fluorescent dyes that allow them to take part in tracking methods such as indocyanine video-angiography and 5-aminolevulinic acid visualization that help monitor blood flow.

There have been a few case examples describing the usage of a 3D exoscope in reference to PNST. Vetrano et al. described a case of a 62-year-old male with a surgical resection of schwannoma of the deep peroneal nerve under the view of a 3D exoscope system, ORBEYE. The procedure was described to have enhanced comfortability compared to the traditional OM utilization, with little need for camera adjustment. The maneuverability allowed enhanced visualization, while the whole surgical team were able to view the same images on a monitor. The real-time anatomy was well visualized in the subsequent video images, while the entire experience was described as natural and nontiring without any visual impairments. The second case report described a 67-year-old male with schwannoma of the sciatic nerve who also underwent surgery using the 3D exoscope view. The OM was again not needed, as the incisor of the left gluteal fold, fascia, and fibers of the biceps femoris were all visualized to reveal the tumor and sciatic nerve [11]. The imaging clearly revealed the difference between tissue bundles and the tumor, as the tumor was well attached to the nerve and required intraoperative neural monitoring. The magnification and focal distance were also described as superior than the conventional OM. The heating effect on the surgical field that occurs with microscopic imaging was also described as absent, indicating the potential usage for treating brain parenchyma [11].

Nevertheless, both cases were able to have the interface between the pathological and surrounding tissue delineated and described, while the instrument was described as an easy and effective optical instrument [11].

Spinal Decompressions and Fusions

3D exoscopes have also been introduced into spinal decompression and fusion surgery. This is commonly performed for patients suffering from lower back pain as a result of degenerative disc disease (DDD). DDD represents the most common physical disability in the United States, with the annual national cost of care reaching over 90 billion dollars [28]. Back and neck pain is commonly attributed to DDD; however, spinal stenosis, disc herniation, and degenerative spondylolisthesis are also common comorbidities of this pathology [28-30]. Specifically, DDD is a reference to the degradation of the intervertebral disc in between the vertebral bodies [31,32]. This occurs due to aging, spinal loading, inflammatory buildup, or genetic predisposition [32]. To correct the subsequent manifestations of DDD, correction surgery such as a decompression and fusion are performed to alleviate the disabling pain [32]. Decompression and fusion are two separate procedures, yet are often combined in this setting to add stability to the affected area [33]. Decompression is a procedure where a surgeon removes a segment of bone, disc, or ligament over a nerve root to alleviate neural pressure that causes pain [34,35]. A fusion is a procedure that structurally joins two segments of a vertebra to add rigidity to the spine or prevent movements that cause pain [34].

In the decompression and fusion surgery, the 3D exoscope is physically smaller and lighter than the traditional OM, allowing for an enhanced comfortable use during the early critical steps of surgery. An example is anterior cervical discectomy and fusion (ACDF) surgery that requires maneuverability of the screw and cage holders [20]. The ability to generate 3D videos that provide the same surgical view and intraoperative image quality enhances the 3D exoscope use for training purposes of conducting an ACDF procedure [2,20]. The 3D exoscope further allows for enhanced illumination and visualization of posterior osteophytes and longitudinal ligaments after removing the intervertebral disc, a key and delicate surgical step that requires application of microsurgical techniques with adequate magnification. The 3D exoscope also makes neural decompressions more straightforward, even among multilevel ACDF cases [2,20].

There are also small case reports that highlight the usage of the 3D exoscope for decompression and fusion surgery. Barbagallo and Certo reported two cases, the first being a 41-year-old female with C6-C7 radiculopathy and the second, a 54-year-old female with neck pain and disc herniation [20]. Both patients underwent successful ACDF surgery under 3D exoscope visualization, with no reported negative outcomes [20]. The 3D exoscope was described to allow for easy maneuvering and adjustment of the surgical field as the posterior osteophytes were effectively removed as the camera tilt allowed for accurate V-shape drilling of the vertebral bodies [20]. The lateral drilling of the posterior medial surface of the uncinated process was also successfully assisted using the 3D endoscope, and was crucial to accomplishing the decompression, as no complications were described. In another study by Siller et al., 40 patients underwent lumbar posterior decompression (LPD) and 20 patients underwent ACDF surgery using the 3D exoscope, and the results were compared to those of the traditional OM [2]. The results showed no significant differences in operative time, blood loss, and postoperative length of stay [2]. Specifically, the operating time was slightly lesser with the 3D exoscope application for the LPD procedures, while the mean length of stay was slightly lower for both LPD and ACDF when using the 3D exoscope. The OM did have lower estimated blood loss in both ACDF and LPD groups; however, none of these parameters achieved statistical significance [2]. The attending surgeon reported enhanced ergonomic comfort in the operating room (OR) when using the 3D exoscope, along with comparable experience in terms of intraoperative instrument handling [2]. However, the surgeon did say that the visualization was slightly superior with the OM in cases of ACDF procedures and long approaches. Specifically, the results showed slightly enhanced depth perception and image quality [2]. Nevertheless, the authors concluded that the 3D microscope is a safe alternative with unique enhancements in comfort that may augment the spinal practice.

Advantages

With respect to visual quality, a 3D exoscope has a capability for 4K resolution or even ultra-HD resolution (depending on the monitor in use). Although lower resolution was originally a limitation of exoscopic systems, currently both 2D and 3D exoscope systems generally have either equivalent or higher resolution than that of an OM [7,9,36-38]. With regard to illumination of the surgical field, the LED light source of an exoscope generates less heat than the halogen bulbs used in OMs [39,40]. This has been reported to be a notable advantage, given it may reduce thermal injury to tissues within the surgical field [39,40]. Another notable advantage is that LED lighting of the exoscope offers a more accurate color contrast of the surgical field [39,40]. This is potentially beneficial to the surgeon, given it allows for an easier recognition of structures when transitioning between micro- and macro-views of the operative field. Perhaps the most significant advantage of 3D exoscope systems is providing a 3D view of the surgical field. Although 2D exoscopes have been noted in the literature to be significantly limited in depth perception, by definition, this limitation does not exist within current 3D exoscopic systems [1,7,9,36,41].

User comfort is a significant and perhaps more practical advantage of the 3D surgical exoscope compared with an OM [9,41-44]. While the user position of an OM requires the surgeon’s neck to be flexed and tensed in certain positions for prolonged periods of time, the exoscope allows for any position or posture to be assumed by the surgeon for the duration of the procedure [41]. Moreover, the comfort benefit also extends to the first assistant, who would otherwise look into the other eyepiece of the OM in a generally uncomfortable position dictated by the primary surgeon [9,37,40]. Additionally, the telescopic design of a 3D surgical exoscope allows for the device to be suspended high above the operative field. This allows for more comfortable maneuverability of surgical instruments during the procedure as well easier communication of the surgeon with the scrub nurse and first assistant due to a clearer path of sight across the operative field [44-46]. The close proximity of an OM to the surgical field is currently a limitation as it commonly blocks both the physical and visual paths across the operative field [37]. This generally conflicts with the comfortable exchange of surgeon’s instruments, prompting need for intra-OR rearrangement around the OM, and may be further complicated depending on the dominant hand of the surgeon. Another benefit is that exoscopes may be sterilized, circumventing the need for sterile cover placement intraoperatively, which is required for OMs [7,36].

Further advantages of a 3D exoscope include potentially more teaching moments and educational benefits that may otherwise be limited. Given an exoscope allows the surgeon to operate while facing the monitor and a large portion of the OR, he or she may be more aware of personnel in the OR and, as a result, be more inclined to teach or invite trainees to watch the procedure [1,43]. The increased openness also allows for easier collaborative efforts to take place between more than one surgeon and allows for more meaningful visual communication with surgical assistants and scrub nurses [38,46-47]. A survey of OR personnel in settings where exoscope use was implemented showed that personnel felt more involved throughout the surgeries and had a greater ability to assist throughout the totality of the procedure when compared with OM use [40]. This benefit also extends to medical students who may be watching. Although OMs may have the capability for monitors to be attached so that those in the OR can watch the microsurgical portions of surgery, viewers in such a setting lack a 3D view of the surgery [8]. Moreover, the surgeon and first assistant are often disconnected from the rest of the OR during OM use due to looking directly into the OM eyepiece. Thus, a surgeon may be unaware of surrounding trainees, leading to missed valuable teaching opportunities. Additionally, a trainee watching an OM monitor is generally unable to concurrently watch the surgeon hands in maneuvering instruments, given the macroscopic view of the surgical field is often blocked by the large OM.

A more recent benefit of exoscopic use was made apparent during the uncertain severe acute respiratory syndrome coronavirus 2 (SARS-CoV-2) pandemic months when the widespread implementation of personal protective equipment (PPE) became a necessity across multiple hospital centers [48]. Barrier precautions with goggles, face shields, or glasses during emergency operations made use of OMs particularly difficult as eyewear had to be removed for the microsurgical portion of the surgeries [48]. Given the significant challenges to surgeons who wished to comply with hospital PPE mandates and maintain barrier precautions to minimize exposure, one hospital in Singapore reported their experience using the Modus V (Synaptive Medical) for emergency spinal trauma procedures [48]. As they were able to retrofit their PPE goggles with the 3D glasses required to utilize the 3D exoscope, this allowed for excellent operative visualization while still maintaining PPE compliance and maximal barrier precautions [48]. Advantages of the 3D exoscope use are further summarized in Table 2.

**Table 2:** Advantages and limitations of exoscope use in neurosurgery 3D: three-dimensional; OM: operating microscope; OR: operating room Source: [7], [9], [36], [43], [48]

Advantages	Limitations
Ergonomic comfort to surgeon (lack of having to hold uncomfortable posture with an OM) - ability to have a relaxed posture with a horizontal gaze versus flexed head and neck position with the OM	Manual reposition of the exoscope during the procedure is difficult and takes longer than with an OM (mitigated with the introduction of robotic stands that allow for foot positioning)
First surgical assistant being able to comfortably assist during the procedure without having to adjust position based on surgeon's positioning of the OM	Learning curve and adaptive period required to develop coordination and familiarity with system
High degree of depth perception	Possible vertigo, headache, or eyestrain from the prolonged use of 3D glasses
Does not consume a large footprint of OR space (compared to OM) due to its compact and lightweight design	Light source sometimes requires additional lighting to achieve a high-resolution image of the operative field
Educational and training opportunities for OR staff, residents, and students	
Ability to see the entire surgical field and rapidly switch from micro- to macro-vision	
Ability for the scrub nurse to participate more actively in procedures	
LED-based illumination generates less heat than the OM with halogen lighting	
4K high definition	
Telescope is distant from the operative field, allowing for greater visualization during the procedure and efficient use of surgical instruments	
Lack of need for lens fogging or cleaning	

Limitations

Given the advanced visual aspects of 3D exoscopes, such as image quality and 3D vision, technical disadvantages are minimal. Initially, the 2D exoscope was introduced and had the significant limitation of lack of depth perception or stereopsis. However, the current 3D capability allows for an excellent depth perception with the use of 3D glasses. A limitation of this aspect of the 3D exoscope has been reports of surgeons potentially developing dizziness, headache, and significant eye strain from the prolonged use of 3D glasses during procedures [8]. Additionally, the aforementioned LED lighting source of exoscopic systems also has a disadvantage as the far distance of the light source from the operative field sometimes requires additional lighting to be introduced to maintain optimal resolution [1,40].

Another disadvantage such as prolonged operative time has been described [7,9,36,43]. This has been attributed to the learning curve that surgeons are confronted with, given the need to develop skills of indirect vision tactics and maneuvering hand movements while watching a monitor that is not in line with their hands [43]. While experienced neurosurgeons may have outstanding hand-eye coordination, the need to coordinate such movements while not looking directly in the plane of movement adds complexity that requires tactic learning [43].

The cumbersome macroscopic re-positioning of an exoscope intraoperatively has also been described as a limitation. The exoscope requires a precise rotational adjustment so that the motions of the surgeons occur in parallel with the orientation of the movements on the screen. Moreover, repositioning an exoscope is currently more complex and time consuming than adjusting an OM, which is equipped with more advanced mechanisms that allow for a comfortable single-handed repositioning. However, this limitation is currently being met with the introduction of robotic exoscopic systems and unlikely to exist as a significant barrier to use. Limitations during exoscope use are further summarized in Table 2.

Cost analysis

While the cost of 3D exoscopic systems has a wide range from $250,000 to $1,500,000 (Table 3), it is generally comparable to the cost of an OM [7,37]. Repair and storage costs may be slightly higher for the OM, given the higher device complexity. Additionally, the continual requirement for sterile covering of the OM during surgery is a considerable cost. Given a disposable sterile drape is estimated to cost $500/drape with a conservative estimation of five procedures/week requiring OM use, the yearly cost to neurosurgical centers just for sterile drapes for an OM is $130,000 [49]. This is considerable compared to exoscopic systems that may be sterilized and thus do not require draping. Thus, although the cost of exoscopic systems is high, neurosurgical centers may consider purchasing an exoscopic system as a potential long-term investment. However, the cost of 3D exoscopic systems is a significant limiting factor to lower budget or community-based hospitals and perhaps neurosurgical departments within developing countries. Additionally, given many of these systems are still first generation, it may be beneficial for such programs to wait until more advanced features of exoscopes become standardized and available across all future models.

**Table 3:** Cost ranges of different exoscopes available *Described by Synaptive Medical as a “microscope” [7,37]

Exoscope System	Approximate Cost (USD)
ORBEYE™ (Olympus, Tokyo, Japan)	~$450,000
KINEVO® (Carl Zeiss Meditec AG, Oberkochen, Germany)	~$900,000
VITOM® 4K 3D (Karl Storz SE & Co. KG, Tuttlingen, Germany)	$250,000-325,000
Modus V™ (Synaptive Medical, Toronto, Canada)*	$600,000-1,500,000

Future directions

Current trends in exoscope development indicate likely future progression in the field of robotic development. Currently, the Modus V exoscope has robot-assisted functions, including the ability for the robotic arm and light source to follow a suction tip, guided by the non-dominant hand, and autofocus on the tip [40,46]. This ability to robotically make otherwise manual adjustments to set precise points of interest into focus has been met with positive reception in several studies [40,46]. Although these studies reported the limitation of poor stereopsis due to 2D exoscope use, Modus V currently has a platform that has robot-assisted movement in addition to 3D visual capability [50]. Studies reporting on this use are currently lacking. The refinement of automated robot-assisted movements and voice-activated functions in a 3D exoscope is likely to improve and continue to revolutionize the neurosurgical user experience.

## Conclusions

The evolution of visual enhancement in neurosurgery has led to the development of the 3D exoscope system, with several clinical studies demonstrating its versatility, safety, and efficacy in a variety of neurosurgical applications. The collection of studies in this review promotes the notion that the 3D exoscope has the ability to provide an immersive optical experience while also being more ergonomically favorable than the OM. In light of these advantages and considering the future direction of the 3D exoscope system, it would be beneficial for future neurosurgical procedures to continue to incorporate this device and report experiences with the revolutionary 3D exoscope.
